# Patients with Newly Diagnosed Cervical Cancer Should Be Screened for Anal Human Papillomavirus (HPV) and Anal Dysplasia: Results of Cost and Quality Analysis

**DOI:** 10.3390/pathogens14101007

**Published:** 2025-10-06

**Authors:** Lukus Berber, Olivia Foy, Jesus Cantu, Eli D. Ehrenpreis

**Affiliations:** Advocate Lutheran General Hospital, Park Ridge, IL 60068, USA; lukus.berber@aah.org (L.B.); olivia.foy@aah.org (O.F.);

**Keywords:** human papillomavirus, anal cancer, cervical cancer, cost analysis, screening

## Abstract

**Background**: HPV infections with high-risk subtypes are a risk factor for developing cervical and anal cancer. Despite HPV vaccination, the incidence of cervical and anal cancer is increasing. There has been substantial research regarding the benefits of screening men who have sex with men (MSM) and those diagnosed with HIV for anal HPV and dysplasia, but little data exists for women diagnosed with cervical cancer. **Methods**: We constructed a Markov model in Python 3.13 to simulate the outcomes and financial impact of screening women newly diagnosed with cervical cancer for anal HPV and dysplasia. Two matrices were used to represent the screened group and the unscreened group. In the screening group, all women received initial anal HPV screening and high-resolution anoscopy with biopsy. If biopsy results confirmed HSIL, each would receive treatment with electrocautery. The screening group would also undergo annual surveillance and follow-up treatment as necessary. In the unscreened group, women did not receive screening or treatment, and the disease process was allowed to progress naturally. **Results**: The initial cohort consisted of 5555 women diagnosed with cervical cancer and concurrent anal HPV. The incidence of anal cancer in the screening group was 271 vs. 375 in the unscreened group after three years, 642 vs. 1236 after ten years, and 863 vs. 2039 after twenty years. Moreover, anal cancer deaths were 1236 in the screening group vs. 9041 in the unscreened group after 10 years and 31,118 vs. 51,553 after twenty years. The screened group saved 330.1 million dollars after ten years and 1.33 billion dollars after twenty years when compared to the unscreened group. Over the life of the study, the screened group would also accrue 102,000 discounted QALYs when compared to the unscreened group. **Conclusions**: Our model strongly suggests that screening women diagnosed with cervical cancer for anal HPV and treating anal dysplasia leads to less anal cancer, less deaths from anal cancer, less economic impact on the healthcare system, and more QALYs for patients.

## 1. Introduction

Human papillomavirus (HPV) is a single-stranded DNA virus from the *Papillomaviridae* family [1]. Many HPV infections are asymptomatic and 90% resolve spontaneously within two years. While typically self-limiting, persistent infection with high-risk HPV subtypes, particularly types 16 and 18, can lead to malignancy, most commonly cervical cancer. It is estimated that 620,000 new HPV-related cancers occur annually throughout the world [2]. Women with cervical HPV infection are approximately seven times more likely to have concurrent anal HPV infection [3]. The International Anal Neoplasia Society (IANS) has classified women with a history of cervical cancer to be at a higher risk for anal cancer [4]. Studies in men who have sex with men (MSM) demonstrate that targeted HPV screening can reduce anal cancer incidence [5]. Women diagnosed with cervical cancer are not routinely screened for anal HPV at this time and we believe this vulnerable population would benefit from further research. Furthermore, as the population ages without the benefit of the HPV vaccine, anal cancer is anticipated to become more common in women older than 65 years of age [6]. Our study uses a cost and quality of life (QOL) model to evaluate the potential benefits of screening for anal HPV and anal dysplasia among women who are newly diagnosed with cervical cancer.

## 2. Methods

We created a dynamic Markov chain model to simulate disease progression and outcomes over 20 years in women newly diagnosed with cervical cancer who either underwent or did not undergo screening for anal HPV. We chose a Markov model as a decision tree to characterize future occurrences based on the present state. Our model was designed to predict future occurrences with reasonable accuracy, such as tumor incidences and deaths, based on assumptions regarding the initial state of the system and the annual rates of change between the various states. [7]. A comprehensive, reproducible literature search with predefined keywords and MeSH terms in PubMed/MEDLINE and related databases were utilized for the model components (initial inputs and annual rates of change between states) for the model (Table 1).

### 2.1. Model Development

**Figure 1:**
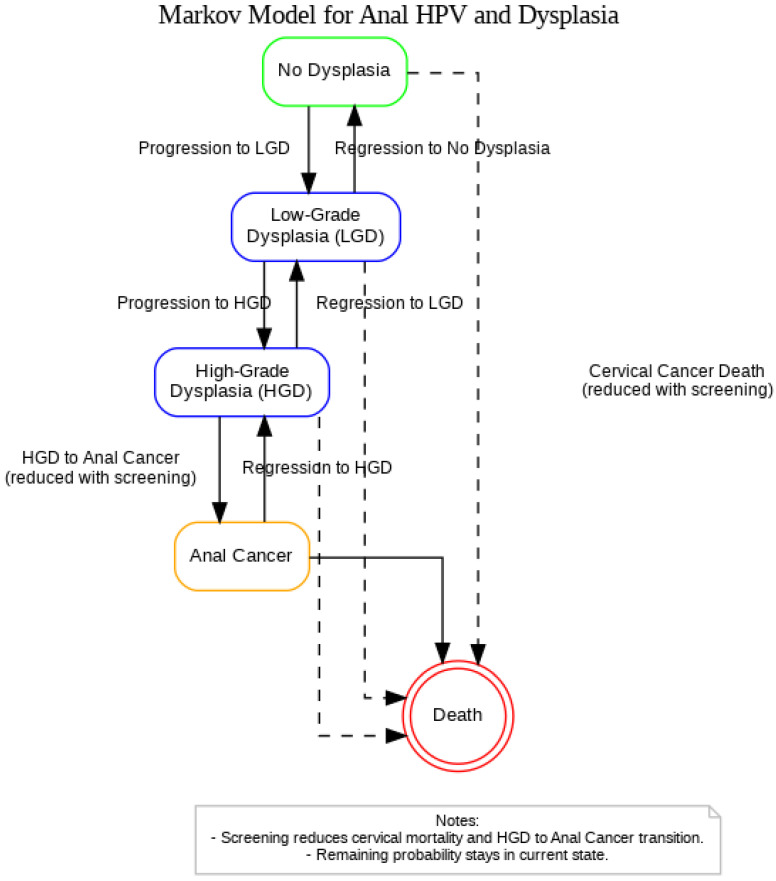
Markov model representing the progression and regression of anal HPV-related dysplasia among women with a history of cervical cancer.

A Markov model was constructed in Python to replicate the STELLA-based simulation originally developed by Ehrenpreis et al. (2018), which evaluated the clinical and economic impact of anal cancer screening in women newly diagnosed with cervical cancer (Figure 1) [21]. The model was reformulated using matrix algebra to simulate annual transitions between health states over a 20-year time horizon. The model includes five mutually exclusive health states: (1) No Dysplasia, (2) Low-Grade Dysplasia (LGD), (3) High-Grade Dysplasia, (4) Anal Cancer, and (5) Death. At each yearly cycle, individuals may remain in their current state, progress to a more advanced state, regress to a less advanced state, or transition to death. Two transition matrices were used: one for a population that receives no screening or treatment (unscreened), and one for a population of women with newly diagnosed cervical cancer that receives screening and treatment for HGD (screened).

Dashed arrows represent cervical cancer-related mortality from any non-cancer state, with risk reduced under screening. Screening is also assumed to reduce the probability of progression from HGD to Anal Cancer. All HPV-positive individuals remain under continuous surveillance and are not modeled to return to an HPV-negative or untested state. The Death state absorbs these cases.

In the screened population, all women undergo initial HPV screening and high-resolution anoscopy with biopsy. If HGD is detected, electrocautery treatment is administered with a base-case cure rate of 95.0%. Individuals in the screened group receive annual surveillance and undergo repeat treatment as needed. In the unscreened population, no screening or treatment is performed, and disease progresses naturally based on previously published progression rates.

The initial model cohort consisted of 5555 women with concurrent cervical cancer and anal HPV infection. This estimate was calculated by applying an anal HPV prevalence rate of 48.3% to 13,820 projected U.S. cervical cancer cases. Baseline distribution across health states was based on anal cytology data from prior studies: 68.0% were placed in the No Dysplasia state, 20.0% in LGD, 9.97% in HGD, and 2.03% in Anal Cancer [22]. No individuals began in the state of death.

At each time point, the distribution of the cohort was updated through matrix multiplication of the current state vector and the corresponding transition matrix. This process was applied for 20 cycles, and annual outputs were recorded. The Python implementation utilized NumPy and Pandas, programs designed to analyze large exponential arrays of data and organize them in data frames, while Matplotlib 3.10.0 and Seaborn 0.13.2 were used to generate visual representations of the data.

### 2.2. Cost Analysis

Cost estimates were obtained from the prior literature and Medicare reimbursement data [19], adjusted to 2025 USD using a 2.05% annual inflation rate [23]. The model included costs associated with HPV screening, cytology, biopsy, HGD treatment, surveillance, and anal cancer management.

Women in the screened group incurred the costs of initial screening, diagnostic evaluation, treatment, and surveillance. Costs were assigned to patients based on their health state each year. In contrast, women in the unscreened group did not incur costs related to screening or treatment of dysplasia treatment found on initial screening but were assigned costs if they developed anal cancer. Total and per-patient costs were calculated for both groups annually and cumulatively over the 20-year simulation horizon.

### 2.3. Sensitivity Analysis

To assess the impact of uncertainty in treatment efficacy, a one-way sensitivity analysis was performed on the cure rate of anal high-grade dysplasia (HGD) following electrocautery. Cure rates varied from 38% to 98%, consistent with published clinical outcomes [22].

For each value, the model estimated cumulative costs and quality-adjusted life years (QALYs) over 5-, 10-, and 20-year time horizons in both screened and unscreened cohorts. Incremental cost-effectiveness ratios (ICERs) were then calculated as the difference in costs divided by the difference in QALYs between the two strategies. Results are reported as cost per QALY gained, allowing for evaluation of cost-effectiveness across a plausible range of treatment efficacies.

### 2.4. Outcomes Determined from the Model

The simulation generated yearly and cumulative estimates for clinically relevant outcomes across the 20-year time horizon. These included the number of new and total cases of anal cancer, deaths attributable to anal cancer, and the aggregate healthcare costs incurred within each group. Model outputs were also used to assess comparative effectiveness by calculating cost per cancer prevented, cost per death averted, and cost per quality-adjusted life year (QALY) gained. These metrics were derived from the longitudinal differences observed between the screened and unscreened populations.

The full range of outcomes produced by the model informed the evaluation of whether routine anal cancer screening and treatment offer a measurable benefit in this high-risk cohort. The rates of histological progression and regression from normal to low-grade dysplasia, high-grade dysplasia, and anal cancer were incorporated into the model [10,11,22]. Some model assumptions were obtained from studies focusing on MSM due to insufficient data in some cases when data in women with cervical cancer were unavailable. Model-generated outcomes included anal cancer incidence, anal cancer-related mortality, quality-adjusted life years (QALYs), total and incremental costs, and cost per QALY gained. QALYs were calculated on a 1-0 scale where 1 is perfect health and 0 represents death. The average quality of life is then multiplied by the years lived after medical intervention to reach QALYs. Our model relies on quality-of-life surveys from cervical and anal cancer patients [5,24]. The overall benefit of the proposed screening program is highly dependent on the effectiveness of curative treatment for anal high-grade dysplasia and therefore a sensitivity analysis was performed for a range of potential cure rates for electrocautery treatment of anal high-grade dysplasia. Outcomes were determined for cure rates of 38%, 48%, 58%, 78%, 88%, and 98%. These factors determined by the simulation performed provide the rationale for screening this patient population. The code for the model and the calculations performed are seen in Supplementary File S1.

## 3. Results

**Figure 2:**
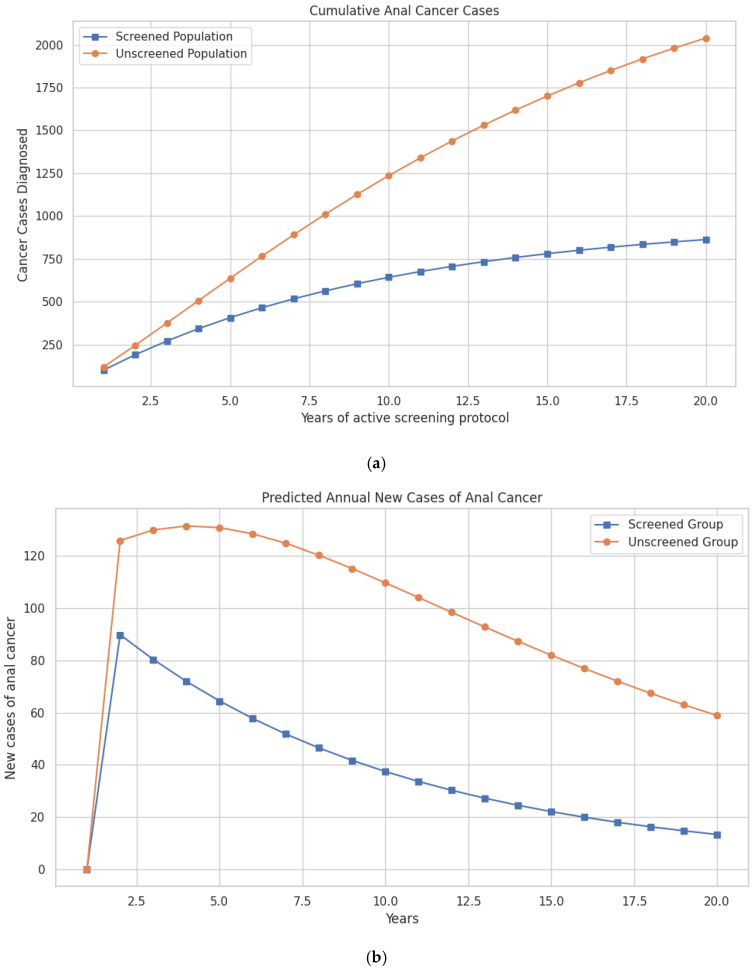
(**a**) Model-predicted cumulative cases of anal cancer over a 20-year period in women with a history of cervical cancer, comparing screened and unscreened populations. (**b**) Model-predicted annual incidence of new anal cancer cases over a 20-year period in women with a history of cervical cancer, comparing those undergoing screening for anal dysplasia with those not screened.

The updated model shows that within the first three years, the difference in cases begins to emerge, with 375 cases in the unscreened group vs. 271 in the screened group. This gap continues to widen over time. By the tenth year, 1236 cumulative anal cancer cases are predicted in the unscreened group vs. 642 cases in the screened group. At 20 years, 2039 cumulative cases occur in the unscreened population compared to 863 cases in the screened population, preventing a total of 1176 cases through screening (Figure 2a; Table 2).

Differences in mortality follow a similar trajectory. Anal cancer deaths are reduced from 16,248 in the unscreened population to 9041 in the screened group at 10 years, and from 51,553 to 31,118 deaths over the 20-year period—representing 20,435 lives saved with screening (Figure 2b; Table 2).

**Figure 3:**
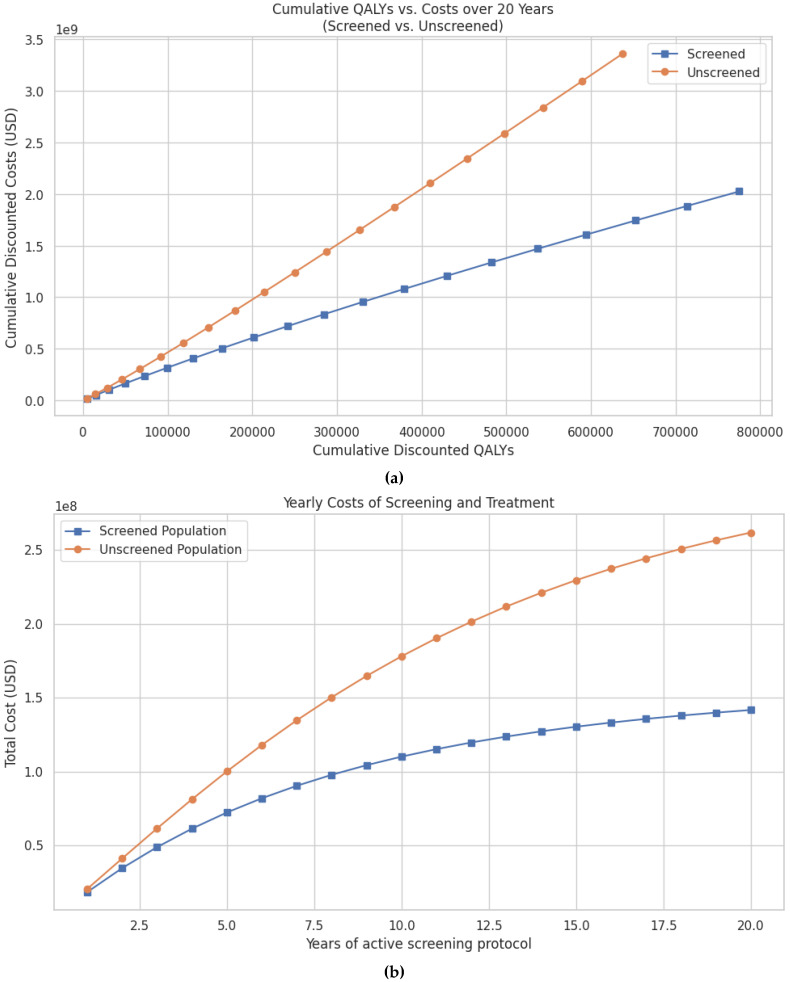
(**a**) Predicted cumulative discounted QALYs vs. cumulative costs over a 20-year period for women with cervical cancer undergoing screening for anal dysplasia compared to no screening. Both QALYs and costs are discounted at an annual rate of 3% to reflect their present value. (**b**) Predicted cumulative costs of screening and treatment for anal cancer over a 20-year period in patients with cervical cancer, comparing screened and unscreened populations. Costs are discounted at an annual rate of 3% to reflect the present value of future expenditures.

There were ~777,000 discounted QALYs compared to ~675,000 QALYs in the unscreened group (Figure 3a). Cumulative discounted costs were also significantly lower in the screened population, totaling USD 2.02 billion vs. USD 3.36 billion in the unscreened group (Figure 3b, Table 3). Thus, screening resulted in savings of USD 1.33 billion and an additional 102,000 discounted QALYs.Per-patient cost-effectiveness outcomes are also favorable. At 20 years, the cost per anal cancer case prevented was USD 109,222, the cost per anal cancer death prevented was USD 7649, and the cost per quality-of-life year saved was USD 9610 (Table 3).

Annual costs also demonstrate early and sustained savings. At 5 years, the screened group had accrued 68.9 million USD less in total costs compared to the unscreened group. These savings increase to USD 330.1 million at 10 years and USD 1.33 billion at 20 years (Table 3).

To assess the robustness of these findings, a one-way sensitivity analysis was performed on the cure rate of anal high-grade dysplasia following electrocautery, ranging from 38% to 98% based on published outcomes. At all tested cure rates and across 5-, 10-, and 20-year time horizons, screening consistently resulted in lower costs and better health outcomes compared to no screening, confirming its status as a cost-saving strategy. For example, at a cure rate of 38%, the cost per QALY gained was USD 2049 at 20 years and improved further to USD 9646 at a 98% cure rate (Table 4).

Although the incremental cost-effectiveness ratios (ICERs) are presented as absolute values in Table 4, all estimates reflect net cost savings with screening. These savings were evident as early as 5 years, where per-QALY costs ranged from USD 3622 to USD 13,234 and became more pronounced over time. Over 10 years, ICERs ranged from USD 3050 to USD 11,909. These results demonstrate that screening remains economically dominant across a wide range of plausible treatment efficacies (Table 4).

## 4. Discussion

The incidence of anal cancer between 2017 and 2021 has increased by 2.9% for women and 1.6% for men [6]. Moreover, according to Cooley et al., cervical cancer deaths are also on the rise in women older than 65 [25]. Screening for cervical as well as anal HPV are considered cost-effective methods to prevent cervical and anal cancer. However, at present, routine screening for anal HPV is only recommended for MSM, patients with HIV, and women diagnosed with vulvar cancer or vulvar HSIL [26]. In 2024, IANS, International Anal Neoplasia Society, made an additional recommendation to include only discretional screening of women with cervical cancer or HSIL [4]. It is important to recognize that while we predominantly draw data related to anal HPV infections from populations of patients with HIV, other immunosuppressed patients have increased risk of developing anal cancer. T-cell surveillance is the primary defense mechanism against dysplasia. Anal and cervical cancer are particularly tied to immunosuppression as both cancers are primarily caused by chronic HPV infection. Chronic HPV infection and failure to clear HPV from infected tissues appear to be the important mechanisms by which HPV causes epithelial dysplasia. A large cohort study in Denmark found that patients with HIV infection, those receiving solid organ transplants, and patients with hematologic malignancies were at higher risk of developing anal cancer [27]. However, patient groups recommended for anal HPV screening are not inclusive for all high-risk groups. For example, women without HIV are not included in screening guidelines for anal HPV and anal cancer [5]. In our view, women newly diagnosed with cervical cancer represent a potential group for cost-effective screening [21].

There is substantial precedence for the use of Markov models in medicine to help reliably predict disease progression, cost effectiveness, and patient outcomes [28,29]. Our modeled screening program suggests there are benefits to screening women with cervical cancer for anal HPV. By conducting minimally invasive anal HPV screening and anal cytology in the setting of HPV infection, fewer women developed anal cancer, fewer women died of anal cancer, and women had a higher quality of life over the time of the study compared to women who were not screened. Moreover, the overall cost of screening and prevention of anal cancer was lower in the screened vs. unscreened group, where treatment of anal cancer, adjusted for inflation [19], superseded screening costs. 

Screening women with cervical cancer for anal HPV meets appropriateness criteria for a screening program; as anal cancer is well understood, it is associated with high mortality, and primary interventions are not prohibitively costly [30]. Sampling of the anal epithelium can easily be performed in an office setting or during endoscopy [31]. Interventions on anal epithelial lesions such as hyfrecation can be completed without general anesthesia and are considered low-risk [12]. Moreover, early interventions on anal epithelial lesions have been shown to reduce the risk of anal cancer [12].

QALYs are a particularly important part of the model as anal cancer treatment is associated with poor quality of life. Chemotherapy and radiation are classically associated with an array of debilitating side effects, but surgical excision for anal cancer can lead to fecal incontinence or even loss of the anus with a diverting colostomy. Anal cancer patients often score poorly on quality-of-life surveys and cite permanent changes to their bodies as the primary reason [32].

The Anal Cancer HSIL Outcomes Research (ANCHOR) study by Palefsky et al. compared treatment of anal HSILs to active monitoring amongst 4459 HIV-positive patients over seven years [22]. The phase three randomized control trial concluded that treatment of anal HSILs was associated with a rate of progression to anal cancer 57% lower than active monitoring with high-resolution anoscopy alone. The literature suggests around 48% of women with high-grade cervical epithelial lesions have concomitant anal HPV infections [12]. There is no study comparable to ANCHOR for women with cervical cancer (an HPV-related cancer), a group at high risk for the development of anal cancer. It is known that 48.3% of women with cervical cancer have anal HPV infection, the majority of these caused by high-risk neoplasm subtypes [12].

When we looked to our colleagues abroad, there is a general consensus, albeit based on limited data in many cases, that screening for anal cancer is not presently recommended with the exception being high-risk groups such as patients living with HIV. Scholefield et al., in the United Kingdom, discussed several limiting factors for screening including the degree of difficulty of identifying histopathology, side effects of intervention on anal lesions, and high recurrence rates after intervention [33]. However, there is promising data that topical cidofovir combined with electrocautery reduced rates of recurrence to zero [33]. Another limiting factor was presented by Li et al., from China, that clinicians skilled in performing high-resolution anoscopy are limited globally [34]. However, both Li and Scholefield mention that some of the data with which we rely for our recommendations is often limited by small sample sizes and unrepresentative patient populations. We agree with our colleagues that the existing data is insufficient, and we believe that patients would benefit from future clinical trials that include women diagnosed with cervical cancer.

It is important to address the limitations of our study. While the data presented in the study is promising, robust clinical trials are required to validate our findings. The data utilized in the model were retrospectively derived from high-quality studies. However, some model assumptions needed to be made from studies of patient groups other than women with cervical cancer. For example, data for the annual progression of anal HPV infection to some epithelial lesions was drawn from a study consisting entirely of MSM and bisexual men in the late 1990s [5] because of the limited existing information on histologic progression in women with anal HPV infections. Moreover, there is data amongst MSM and patients with HIV that indicates a high recurrence rate: 53% recurrence amongst HIV-negative patients and 61% amongst patients with HIV [35]. Our model assumes a 95% cure rate with intervention, which is appropriate given our model cohort has functioning immune systems, but does not presently address recurrent dysplasia or the requirement for repeat interventions. While Marks et al. does not include women diagnosed with cervical cancer specifically, which our model considers directly, it does provide a route for further analysis in the future [35]. Additionally, the literature suggests that while recurrence of dysplasia does occur after intervention, development of anal cancer thereafter is quite rare—around 1% [36]. The lack of women-focused data does, however, illuminate a core premise of this study—our model suggests women at high risk for anal HPV infection should be primary targets for future research. A fundamental assumption in this study is that chronic anal HPV infections with high-risk HPV subtypes lead to progression from LGD, then to HSIL, and finally to anal cancer. While it is known that extended activity of HPV oncogenes leads to DNA mutations and ultimately anal cancer [37], progression of epithelial lesions may follow a different pattern than our assumption in the model.

In summary, when women with a new diagnosis of cervical cancer are screened for anal HPV, evaluated for anal epithelial dysplasia, and treated for anal HSIL, they are less likely to develop anal cancer, less likely to die from anal cancer, and have a higher quality of life. Our model also predicts that the proposed screening program is cost-effective as screening saves an estimated cost saving per diagnosed anal cancer of USD 120,416 at 5 years, USD 117,506 at 10 years, and USD 109,922 at 20 years. The model strongly suggests that providers should be cognizant of the risk of anal HPV in women diagnosed with cervical cancer and investigate accordingly.

## Figures and Tables

**Table 1:** Model assumptions.

Annual incidence of cervical cancer in the US attributable to HPV	13,820 [2]
% of women with a history of cervical high-grade dysplasia or microinvasive cancer with anal HPV	48.3% [8]
Total number of women with a history of cervical high-grade dysplasia or microinvasive cancer with anal HPV	5550
Average age at time of diagnosis of cervical cancer	50 [9]
% of women with a history of genital cancer initially having anal ASC-US	19.4% [10]
% of women with a history of cervical cancer initially having no anal dysplasia	50.8% [10]
% of women with a history of high-grade cervical cytology initially having anal low-grade dysplasia	16% [10]
% of women with a history of genital cancer initially having anal high-grade dysplasia	3% [10]
% of women with anal HPV and no dysplasia that develop high-grade dysplasia over two years	8% [11]
% of women with anal HPV and low-grade dysplasia that develop high-grade dysplasia over two years	36% [11]
% of women with anal HPV and ASC-US that develop high-grade dysplasia over two years	62% [12]
ACUS cytology equivalents (based on 1-year follow-up cytology in MSM)	ACUS represents normal = 58%, ACUS represents LGD = 24%, ACUS represents HSIL = 18%, ACUs represents cancer = 0 [5]
Histological progression and regression (based on 1-year follow-up in MSM)	Progression: Normal to LGD = 1.9%, Normal to HGD = 1.78%, Normal to cancer = 0%, LGD to HGD = 16.5%, LGD to anal cancer = 0.05% (estimated), HGD to anal cancer = 3.6% Regression: LGD to normal = 22.65%, HGD to LGD = 22% (estimated), HGD to normal = 11.36% [5,13]
Annual anal cancer death rate	8.96% [14]
Five-year anal cancer death rate	29.4% [14]
Annual death rate from cervical cancer	11.73% [15]
Five-year death rate from cervical cancer	32.6% [15]
Average life expectancy for a female age 49	32.95 [16]
Quality of life weight adjustment for anal cancer	0.56 [5]
Quality of life weight adjustment after treatment for anal HGD	0.9 [5]
Quality of life weight adjustment for cervical cancer	0.7 [17]
Quality-adjusted life expectancy woman age 49	21.6 [18]
Quality-adjusted life expectancy woman age 49 surviving cervical cancer	21.6 × 0.7 = 15.12
Quality-adjusted life expectancy woman age 49 surviving cervical cancer treated for anal HGD	15.1 × 0.9 = 13.6
Cost of HPV screen *	USD 104 [19]
Cost of anal cytology *	USD 72 [19]
Cost of treatment of anal HGD *	USD 281 [19]
Cost of high-resolution anoscopy and biopsy *	USD 288 [19]
Cost of anal cancer treatment *	USD 150,000 [20]

* 2025 Costs are annualized with estimated 2.05% inflation rate in the model.

**Table 2:** Model estimates of cumulative anal cancers and anal cancer deaths in cervical cancer patients that are screened and treated or not screened and treated for anal HPV and dysplasia.

Year	Anal Cancers (Unscreened)	Anal Cancers (Screened)	Cancer Deaths (Unscreened)	Cancer Deaths (Screened)
1	119	101	356	182
2	295	199	1045	399
3	373	271	2036	1066
4	507	343	3338	1706
5	637	407	4704	2605
6	791	460	5787	3476
7	891	505	6787	4755
8	1011	563	11,076	6048
9	1136	603	13,657	7428
10	1236	646	16525	9074
11	1340	676	19,179	10751
12	1438	702	21,919	12,954
13	1510	729	24,729	14,984
14	1618	758	28,744	16,544
15	1740	780	29,744	18,614
16	1817	803	33,147	20,039
17	1919	830	37947	22967
18	1987	855	43,447	25,856
19	1809	879	48,987	28,439
20	2039	863	51,553	31,115

**Table 3:** Estimates of costs from dynamic modeling for screening for HPV and treatment of anal dysplasia in patients with cervical cancer.

	Costs in Unscreened Group (Cancer Care Only)	Costs in Screened Group (HPV Screening, Annual Cytology, Treatment of HSIL, and Cancer Care)	Cost Difference	Cost per Anal Cancer Prevented	Cost per Anal Cancer Death Prevented	Cost per Quality-of-Life Year Saved
**5 years**	USD 304,498,048	USD 235,563,777	USD 68,934,271	USD 120,416	USD 12,438	USD 12,926
**10 years**	USD 1050,234,416	USD 720,133,360	USD 330,101,055	USD 117,506	USD 10,640	USD 11,830
**20 years**	USD 3355,123,274	USD 2024,718,550	USD 1330,404,723	USD 109,222	USD 7649	USD 9610

**Table 4:** Results of one-way sensitivity analysis showing the incremental cost per quality-adjusted life year (QALY) gained at varying cure rates of anal high-grade squamous intraepithelial lesions (HSIL) treated with electrocautery.

Cure Rate of Anal High-Grade Dysplasia	Cost per QALY Gained 5 Years	Cost per QALY Gained 10 Years	Cost per QALY Gained 20 Years
0.38	USD 3622.22	USD 3049.97	USD 2049.48
0.48	USD 5229.36	USD 4514.67	USD 3289.81
0.58	USD 6834.47	USD 5984.13	USD 4540.34
0.78	USD 10,038.51	USD 8937.30	USD 7072.34
0.88	USD 11,637.40	USD 10,420.96	USD 8354.02
0.98	USD 13,234.15	USD 11,909.33	USD 9646.29

## Data Availability

The original contributions presented in this study are included in the article/Supplementary Materials. Further inquiries can be directed to the corresponding author.

## Supplementary Materials

The following supporting information can be downloaded at: https://www.mdpi.com/article/10.3390/pathogens14101007/s1, File S1: Replication of the Ehrenpreis Cost Model for Anal HPV (2025 Update).
